# Does the surgical route matter in cerebellar mutism syndrome? Comparing transvermian and telovelar approaches for pediatric posterior fossa tumors

**DOI:** 10.1016/j.bas.2026.106168

**Published:** 2026-07-27

**Authors:** Merle Egeborg, Rebekka Sarup, Marianne Juhler, Aske Foldbjerg Laustsen

**Affiliations:** a Department of Neurosurgery, Rigshospitalet, Copenhagen, Denmark; b Department of Pediatrics and Adolescent Medicine, Rigshospitalet, Copenhagen, Denmark; c Department of Neurosurgery, Aarhus University Hospital, Aarhus, Denmark

**Keywords:** Cerebellar mutism syndrome, Pediatric neurosurgery, Posterior fossa syndrome, Posterior fossa tumor, Postoperative speech impairment, surgical approach

## Abstract

**Introduction:**

Children and adolescents undergoing surgery for posterior fossa brain tumors are at risk of developing cerebellar mutism syndrome (CMS). Mutism or reduced speech is a cardinal symptom; however, CMS is also characterized by motor, cognitive, and emotional difficulties that may persist as long-term sequelae. The potential association between vermian incision during tumor resection and an increased incidence of CMS remains a subject of debate in the literature.

**Research question:**

This review aimed to evaluate whether the telovelar approach is associated with a lower risk of CMS compared with the transvermian approach.

**Material and methods:**

Original studies were identified through a structured literature search of the PubMed and EMBASE databases. Postoperative speech outcomes and surgical approaches were extracted, and a pooled odds ratio (OR) was estimated using a random-effects model.

**Results:**

16 studies (PS = 6, RS = 9, mixed PS/RS = 1) met the inclusion criteria, comprising a total of 1369 patients. A pooled estimate yielded an OR of 1.85 (95% CI: 0.95-3.59, *p = 0.07*). Subanalysis of prospective studies yielded a pooled OR of 1.10 (95% CI: 0.29-4.19) and did not alter the overall result.

**Conclusion:**

This review adds to the existing evidence on surgery-related risk factors for CMS. We found no significant difference in CMS risk between transvermian and telovelar approaches, indicating no clear advantage of either approach in preventing CMS.

## Highlights

• Conflicting evidence on transvermian approach and CMS in the literature.
• This meta-analysis found no significant increase in CMS risk with vermal incision.
• Telovelar approach is not superior in preventing cerebellar mutism syndrome.
• Cerebellar mutism syndrome likely depends on factors beyond surgical approach.

## Abbreviations

CI: Confidence interval
CM: Cerebellar mutism
CMS: Cerebellar mutism syndrome
Log OR: Log odds ratio
MC: Multicenter
MSD: Cerebellar mutism and subsequent dysarthria
OR: Odds ratio
PFS: Posterior fossa syndrome
PICO: Population, intervention, comparison, outcome
POSI: Post-operative speech impairment
PRISMA: Preferred reporting items for systematic reviews and meta-analyses
PS: Prospective study
RS: Retrospective study
SC: Single-center
SE: Standard error

## 1 Introduction

Cerebellar mutism syndrome (CMS) is a recognized complication to posterior fossa tumor surgery in pediatric patients, with an incidence of up to 30% (Grønbæk et al., 2021). It typically presents within a few days postoperatively, with mutism or reduced speech as the cardinal symptom. While many patients gradually recover, up to 25% of the affected children do not regain their speech abilities within 80 days after symptom onset (Grønbæk et al., 2021). CMS is also frequently accompanied by motor, cognitive, and emotional impairments that may persist as long-term sequelae, thereby negatively impacting quality of life throughout the lifespan (Fabozzi et al., 2022; Wibroe et al., 2021).

Historically, CMS has not been clearly defined or delineated, and numerous terms in addition to CMS have been used to describe similar symptoms(Grønbæk et al., 2021; Catsman-Berrevoets et al., 1999; de Laurentis et al., 2022; SeveroBem et al., 2021; Wisoff and Epstein, 1984). CMS has gained increasing attention over time, and in response to the variability in terminology and definitions, a consensus paper on ‘‘post-operative pediatric cerebellar mutism syndrome’’ was published in 2016 (Gudrunardottir et al., 2016).

It is widely agreed that preoperative risk factors for CMS include younger age, midline tumor location, medulloblastoma histopathology and brainstem invasion, but the underlying pathogenesis has yet to be fully understood (Pettersson et al., 2022). Surgical injury to the dentato-thalamo-cortical pathway (DTCP) has been suggested as an important factor contributing to the development of CMS (Avula et al., 2015). Traditionally, the cerebellum was primarily attributed to motor functions, but its role in higher brain functions has gradually been recognized(Rudolph et al., 2023). As part of a circuit between the cerebellum and cerebrum, DTCP has been considered crucial for linguistic, motor and cognitive functions (Avula et al., 2015; Toescu et al., 2018). Recent studies suggest additional explanations to the pathophysiological mechanisms of CMS, emphasizing the role of the fastigial nucleus and the periaqueductal gray matter (McAfee et al., 2023a, 2023b, 2024).

Many studies have investigated whether the incidence of CMS can be influenced by modifiable surgery-related factors. Different surgical approaches, particularly the transvermian and the telovelar approaches, have been frequent topics of discussion. These surgical routes are used to access the fourth ventricle and posterior fossa midline, which is not only a high-risk area for CMS, but also the site where most pediatric posterior fossa tumors are located (Pettersson et al., 2022; Wells and Packer, 2015).

In this study, we aimed to systematically review the existing literature to evaluate whether the telovelar approach is preferable to the transvermian approach in reducing the risk of CMS.

## 2 Materials and methods

### 2.1 Search strategy

A literature search was conducted in PubMed and EMBASE. The detailed search strategy is available in supplementary material. The search was performed on September 10, 2024.

The literature search was structured with a PICO framework: this review included children and adolescents (<18 years of age at diagnosis) undergoing tumor resection in the posterior fossa, patients were grouped based on whether the surgical approach involved an incision to the cerebellar vermis (e.g., ‘‘transvermian approach’’ or ‘‘vermal incision’’) or with an approach presumed to minimize vermian injury (e.g., ‘‘telovelar approach’’ or ‘‘lateral to vermis approach’’) and the outcome of interest was CMS or mutism. Only prospective, retrospective, or mixed observational cohort studies were included. Full-text availability in English was another requirement. Precalculated odds ratio (OR) or data enabling OR calculation had to be available for data extraction. Studies were excluded if only a single surgical approach was used for all patients, as our primary aim was to compare different access routes in relation to the risk of CMS, which required stratification by surgical approach. Furthermore, studies including secondary or multiple surgeries were excluded to prevent patients from being included more than once in the analysis. Mixed cohorts including both children and adults were excluded when data specific to the pediatric population could not be extracted.

### 2.2 Screening and full-text review

Title and abstract screening, full-text review, and data extraction were conducted independently by two reviewers in PubMed and EMBASE (A.F.L and R.S.). Quality assessment was also performed independently by the same two reviewers. Any conflicts were resolved through discussion to reach consensus, and unresolved disagreements would have been referred to a third reviewer (M.J.); however, this was not necessary.

### 2.3 Data extraction

The names of the first authors, publication date, cohort inclusion period, country of origin, study design, cohort size, patient characteristics (tumor histology and location), prevalence, terminology for surgical approach and speech outcome were extracted. A 2 x 2 matrix of surgical approach and speech outcome was extracted for OR calculation.

### 2.4 Quality and publication bias assessment

The quality of the included studies was assessed using a modified Newcastle-Ottawa Scale(Wells et al., 2000). The scale consists of three categories with a maximum of 4 points assigned for the *selection* category, 1 point for *comparability*, and 3 points for the *outcome* category. A score of ≥7 was considered high quality, 4-6 as moderate quality, and ≤3 as low quality. Publication bias among the included studies was assessed using funnel plot asymmetry *(Supplementary material,* Fig. S3*).*

### 2.5 Statistical analysis

Unadjusted OR with 95% confidence intervals (CI) from the included studies were pooled using a random-effects model with the inverse variance method. DerSimonian-Laird was used as tau^2^ estimator, the Jackson method for confidence interval of tau^2^ and tau, and Hartung-Knapp adjustment for random effects model (df = included studies minus 1). A continuity correction of 0.5 was applied in studies with zero cell frequencies. Heterogeneity was assessed with the I^2^ statistics. Statistical analyses were performed in R (v.4.4.1) using the *meta* and *ggplot2 packages*.

## 3 Results

### 3.1 Literature search

**Fig. 1:**
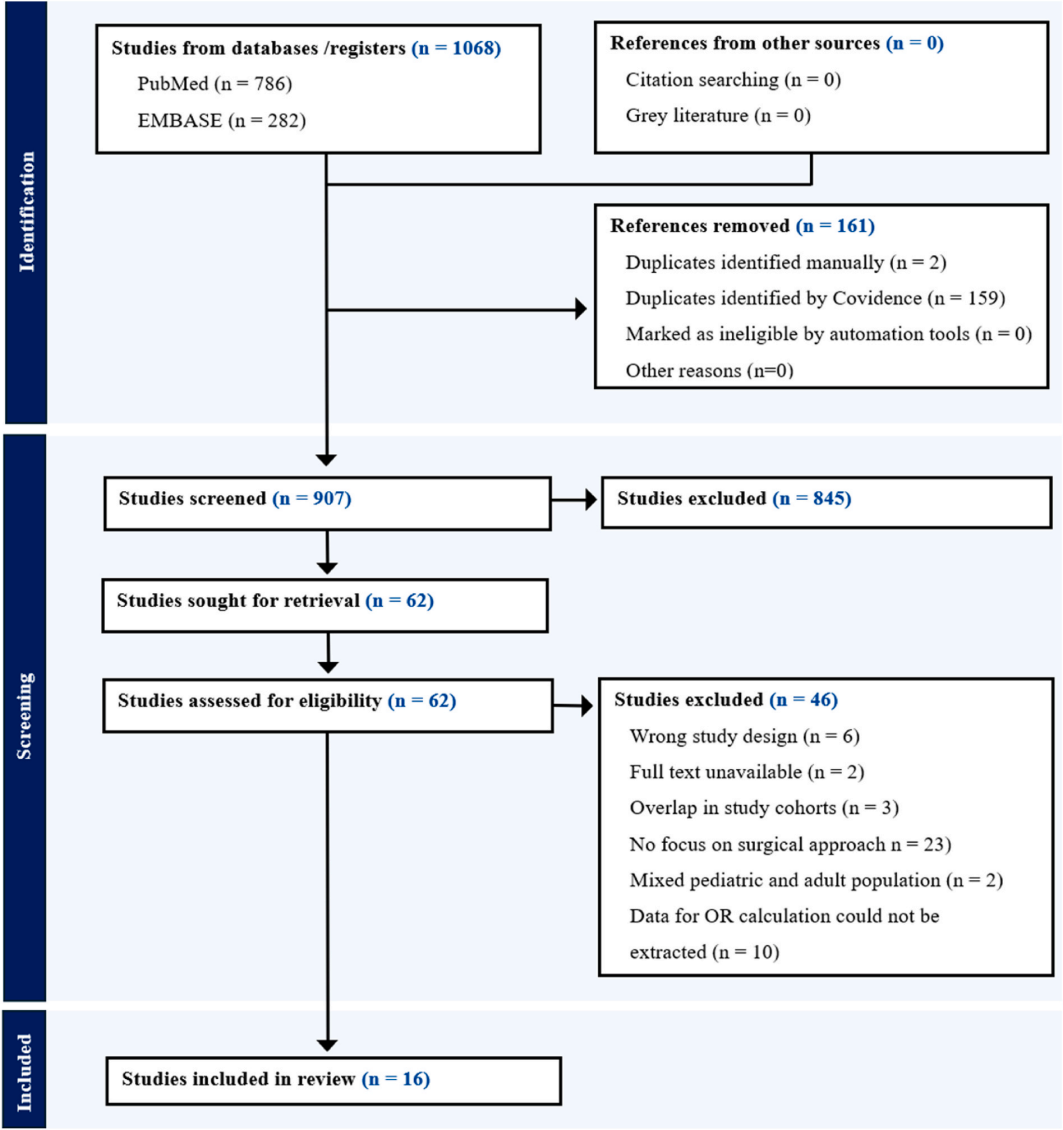
PRISMA flowchart showing study identification through database searches.

The title and abstract screening, followed by full text assessment, resulted in 16 studies meeting the inclusion criteria. The screening and review process is summarized in a PRISMA flowchart (Fig. 1).

### 3.2 Study exclusion

Four studies met PICO inclusion criteria but were excluded from this review because the data necessary for meta-analysis could not be extracted. One of these was a retrospective international multicenter study (n = 263) that did not find a significant association between CMS and surgical approach(Renne et al., 2020). Another was a retrospective single-center study (n = 63) reporting that the association between CMS and surgical approach became non-significant in multivariate analysis(Korah et al., 2010). Additionally, a retrospective study (n = 28) found an association between vermal dissection and increased risk of CMS and a retrospective study (n = 31) described vermal incision as a perioperative risk factor for CMS(Wells et al., 2010; Aarsen et al., 2022).

### 3.3 Study characteristics

CMS incidence ranged from 7,9% to 50,0% (Table 1). Only two studies presented OR in the original articles, and data from all studies were therefore extracted for unadjusted OR calculation (Grønbæk et al., 2021; Onorini et al., 2022). Extracted data resulted in zero frequencies in three studies, for which a continuity correction of 0.5 was applied in the primary meta-analysis (Table 2). Study outcomes of ‘‘cerebellar mutism’’, ‘‘cerebellar mutism syndrome’’, ‘‘posterior fossa syndrome’’, ‘‘mutism and subsequent dysarthria’’, ‘‘mutism’’ and ‘‘postoperative speech impairment’’ were considered comparable and collectively labelled as CMS in the analysis.

**Table 1:** Study characteristics.

Author	Year	Cohort inclusion period	Origin	Study design	Cohort size	Tumor histology	Tumor location	Terminology for surgical approach	Terminology for outcome	Prevalence	Summary of findings	Quality
**Catsman-Berrevoets et al.**	1999	-	The Nether-lands	PS, SC	42	Multiple	Cerebellum	Vermal incision/Incision lateral to vermis	MSD	29%	Vermal incision was identified as a significant risk factor (p-value = 0,028)	High
**Cobourn et al.**	2019	2000 –2016	USA	RS, SC	65	Medullo-blastoma	Posterior fossa	Vermal incision/No vermal incision	CMS	10,8%	Vermal incision was identified as a significant risk factor (p-value = 0,03)	Moderate
**De Laurentis et al.**	2022	2003 –2019	Italy	RS, MC	109	Medullo-blastoma	Posterior fossa	Intraoperative involvement of vermis/No intraoperative involvement of vermis	PFS	27%	Intraoperative involvement of the vermis was associated with CMS, but the association was not statistically significant	High
**De Smet et al.**	2011	1995 – 2007	Belgium	RS, SC	24	Multiple	Posterior Fossa	Vermal incision/No vermal incision	Mutism	50,0%	An increased rate of mutism was observed when the vermis was incised	High
**Di Rocco et al.**	2011	2006 –2008	Italy	PS, SC	34	Multiple	Posterior fossa	Vermis split/No Vermis split	CMS	20,6%	Vermis split was not associated with CMS	High
**Doxey et. al.**	1999	1985 – 1998	USA	RS, SC	253	Multiple	Posterior fossa	Vermal incision/No Vermal incision	PFS	7,9%	The vermis was incised in all 20 patients who developed mutism. 98 of the 253 children had the vermis incised without development of CMS	Moderate
**Erşahin et al.**	2002	1998 –2000	Turkey	PS, SC	14	Multiple	Posterior fossa	Vermal incision/No Vermal incision	Mutism	21,4%	Vermal split was not a significant risk factor.	Moderate
**Gora et al.**	2017	2015 –2016	India	PS, SC,	33	Multiple	Posterior fossa	Vermal incision/No Vermal incision	CMS	18,2%	Incision to the middle part of the vermis was significant risk factor (p-value = 0,007)	High
**Grill et al.**	2004	1996 –2001	France	RS, SC	76	Multiple	Posterior fossa	Vermal incision/No Vermal incision	CM	11,8%	The vermis was incised more often in CMS patients (p-value = 0,03)	Moderate
**Grønbæk et al.**	2021	2014 –2021	Europe	PS, MC	376	Multiple	Posterior fossa	Transvermian/Telovelar	POSI	14% (Mutism) 16% (Reduced speech)	Surgical approach was not a significant risk factor.	High
Haratek et al. (2023)	2023	2006 –2021	Czech Republic	RS, SC	106	Multiple	4th ventricle	Transvermian/Telovelar	CMS	20,8%	Surgical approach was not a significant risk factor	Moderate
**McMillan et al.**	2009	1990 –2007	Canada	RS, SC	51	Multiple	Midline, Posterior fossa	Vermal incision/No vermal incision	Mutism	25%	There was no difference in the rate of vermal incision between the mutism and no-mutism groups	Moderate
**Onorini et al.**	2022	2007 –2018	Italy	RS, SC	92	Multiple	4th ventricle	Vermal incision/No Vermal incision	CMS	11%	CMS was not associated with any surgical approach.	Moderate
Schmidt et al. (2023)	2023	2010 –2021	Germany	RS, SC	60	Multiple	Posterior fossa	Transvermian/Telovelar	CMS	17%	Transvermian approach was a significant risk factor (p-value = 0,03).	High
**Toescu et al.**	2020	2002 –2018	UK	PS, SC	167	Multiple	4th ventricle	Transvermian/Telovelar	CMS	28,7%	Surgical approach was not a significant risk factor	High
**Yang et al.**	2022	2013 –2021	China	Mixed (PS and RS), SC	218	Multiple	Posterior fossa	Transvermian/Telovelar or other approach	CMS	32,6%	No significant difference was found between the transvermian and the telovelar approach in the risk of CMS	High

Study-type.

PS: prospective study, RS: retrospective study, SC: single-center, MC: multicenter.

Terminology.

MSD: cerebellar mutism and subsequent dysarthria, CM: cerebellar mutism, CMS: cerebellar mutism syndrome, PFS: posterior fossa syndrome, POSI: post-operative speech impairment.

**Table 2:** Data extracted for meta-analysis.

Author	Vermal incision		No vermal incision	
	CMS	Non CMS	CMS	Non CMS
**Catsman-Berrevoets et al.**	10	12	2	18
**Cobourn et al.**	6	26	1	32
**De Laurentis et al.**	13	19	16	61
**De Smet et al.**	11	8	1	4
**Di Rocco et al.**	1	10	6	17
**Doxey et. al.**	20	98	0	135
**Erşahin et al.**	2	10	1	1
**Gora et al.**	6	14	0	13
**Grill et al.**	7	31	2	36
**Grønbæk et al.**	30	59	50	73
**Haratek et al**	14	60	8	24
**McMillan et al.**	11	30	0	3
**Onorini et al.**	6	35	4	47
**Schmidt et al**	3	2	4	22
**Toescu et al.**	29	78	17	38
**Yang et al.**	17	24	15	16

The results of the included studies were conflicting regarding whether CMS is associated with the use of a transvermian approach. Seven studies reported an association between the transvermian approach and an increased risk of CMS(Catsman-Berrevoets et al., 1999; Cobourn et al., 2020; De et al., 2012; Doxey et al., 1999; Gora et al., 2017; Grill et al., 2004; Schmidt et al., 2023), while the remaining nine studies did not find evidence to support this association (Table 1) (Grønbæk et al., 2021; de Laurentis et al., 2022; Onorini et al., 2022; Di Rocco et al., 2011; Erşahin et al., 2002; Haratek et al., 2023; McMillan et al., 2009; Toescu et al., 2021; Yang et al., 2023). Data extraction resulted in 1369 patients included in the meta-analysis (Table 2).

### 3.4 Quality and publication bias assessment

A total of nine studies were assessed as high quality, seven as moderate quality, and none as low quality (Table 1 and Supplementary Table S1). Quality ratings were based on the information available on surgical approach and speech outcome and are not representative of the overall methodological quality of the studies. It should be noted that only unadjusted ORs extracted from the studies were included in our meta-analysis, as multivariate analyses across the studies varied in methodology and did not account for the same confounders.

Funnel plot assessment indicated asymmetry, with a higher number of published studies reporting negative LogOR values and lower standard errors, alongside a concentration of studies with positive LogOR values and higher standard errors. The funnel plot asymmetry demonstrates that studies showing a positive LogOR tend to have a retrospective design (Supplementary Fig. S3).

### 3.5 Meta-analysis

**Fig. 2:**
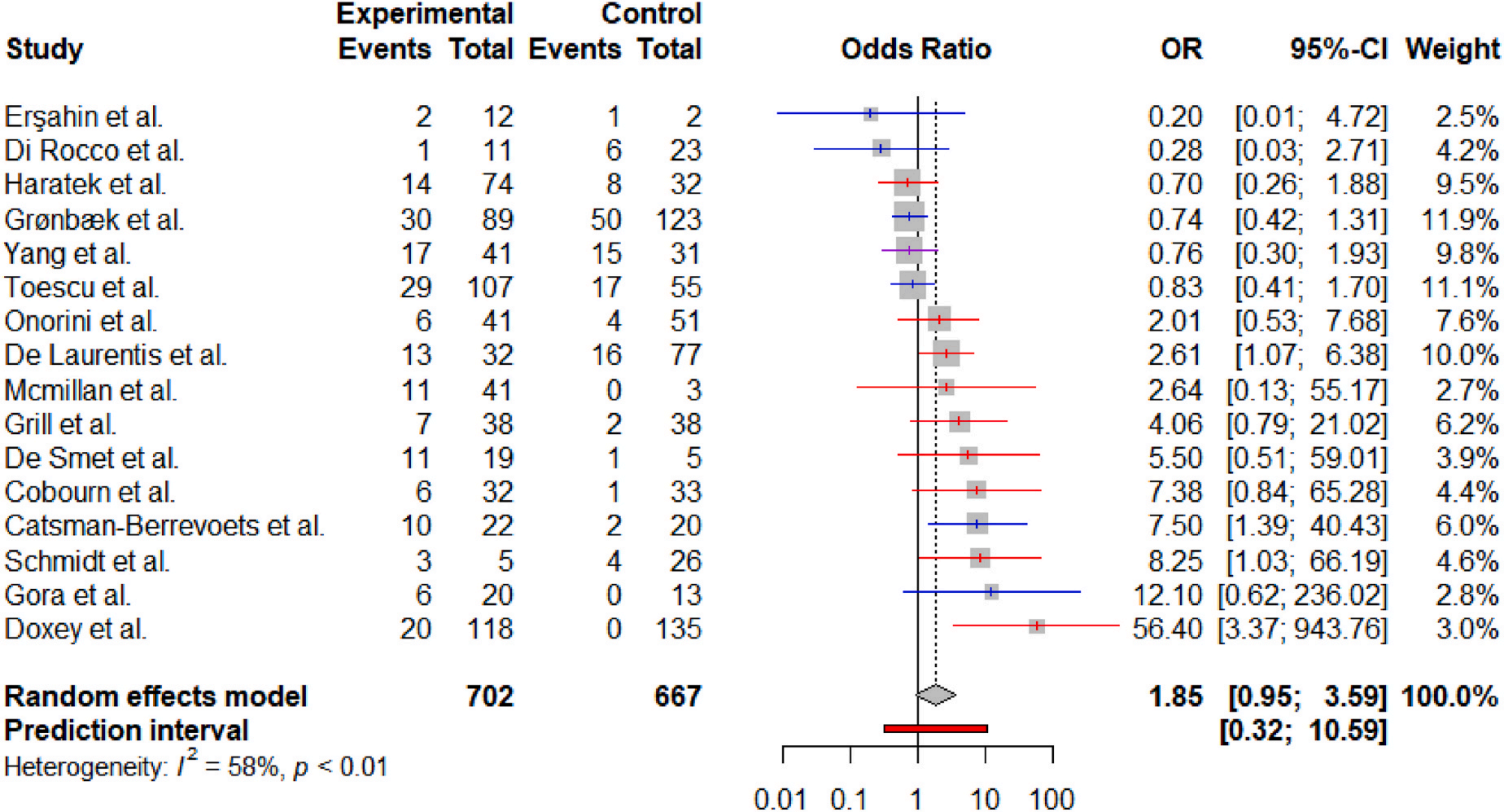
Forest plot of all included studies Interpretation of odds ratio: **OR = 1** indicates equal odds of CMS in both groups. **OR > 1** indicate increased odds of CMS when the vermis is incised during surgery (i.e’’transvermian approach’’) compared to when the vermis was reportedly spared (i.e ‘‘telovelar approach’’). **OR < 1** indicates that transvermian approach does not increase the odds of CMS; but it shoud not be interpreted as a definitive reduction in risk. A **wide confidence interval (CI)** reflects uncertainty in the OR estimate. If the **CI crosses 1** the observed effect is not statistically significant, since a OR = 1 corresponds to no difference between the two groups. Colors in the plot represent different study designs: red = retrospective, blue = prospective, and purple = mixed cohort of prospective and retrospective.

**Fig. 3:**
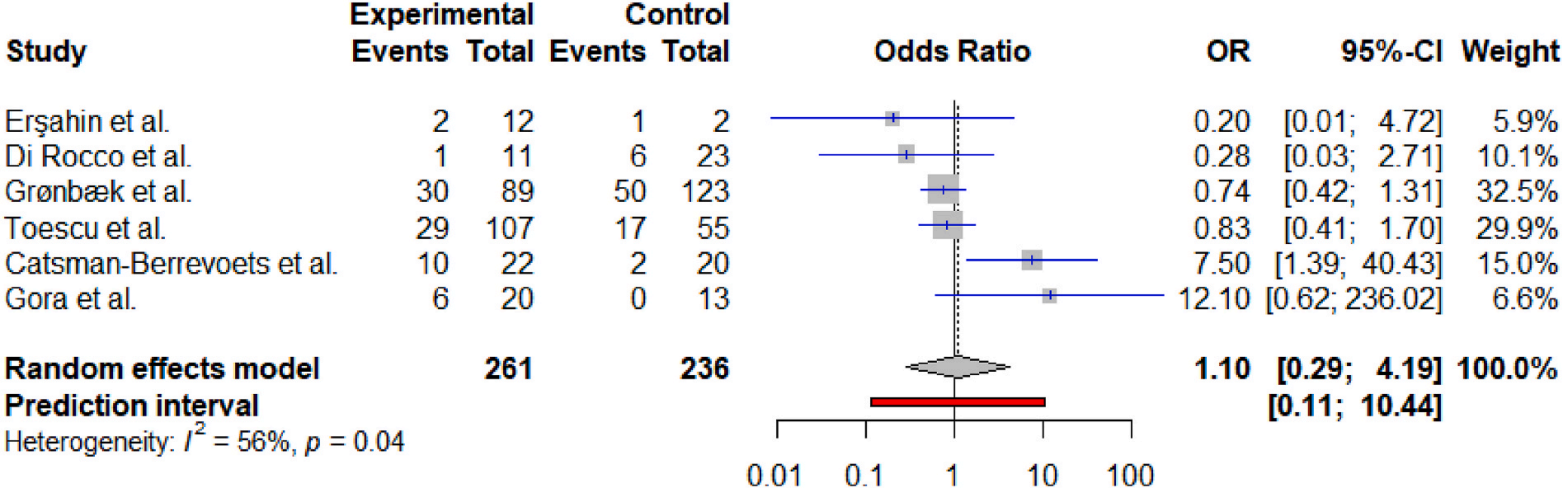
Forest plot showing only prospective studies.

As seen in Fig. 2, individual studies demonstrate variability in effect estimates, with OR distributed on both sides of unity and there is no consistent pattern across studies. The pooled estimate of all included studies was OR 1.85 [95% CI: 0.95-3.59, *p = 0.07*]. Since the CI crosses 1 and P > 0,05 this is interpreted as non-significant. The between-study heterogeneity variance was estimated to be tau^2^ = 0.58 [95% CI: 0.15-3.30], with an I^2^ value of 58% indicating moderate study heterogeneity. Greater heterogeneity makes it difficult to detect a statistically significant overall effect, as the study results are not consistently concordant. The prediction interval ranged from g = [0.32; 10.59] and this wide interval indicates that a future study of a similar design is unlikely to yield a clear effect and therefore are unlikely to favor one surgical approach over the other. Funnel plot asymmetry revealed a moderately right-skewed publication bias (Supplementary Fig. S3). This indicates that the meta-analysis may be influenced by publication bias (due to the absence of small studies with weak or negative results on CMS risk in relation to transvermian approach). A subanalysis excluding studies with zero values slightly reduced the overall OR and did not substantially alter the results (Supplementary Fig. S4). Due to the potential bias of retrospective studies, a sub-analysis including only prospective studies (n = 6) was also performed. The pooled effect estimate of this sub-analysis was OR 1.10 [95% CI: 0.29-4.19] and not statistically significant (Fig. 3).

Interpretation of the odds ratio: see under Fig. 2.

## 4 Discussion

By providing an updated synthesis of the available evidence, this review contributes to the ongoing debate on the role of surgical approach in the development of CMS. In our meta-analysis, a transvermian approach was not associated with a statistically significant increase in CMS risk, suggesting that surgical approach alone may be an imprecise predictor of CMS. This is consistent with a recent systematic review, which similarly found no significant association between surgical approach and the risk of various postoperative complications, including CMS, following resection of fourth ventricular tumors (Hanaei et al., 2024). Although earlier small retrospective studies have suggested an increased risk with a transvermian approach, this has not been consistently supported by more recent studies with larger sample sizes.

### 4.1 Strengths and limitations

#### 4.1.1 Absence of randomized controlled trials

A limitation of this analysis is the absence of evidence from randomized controlled trials. To our knowledge, no randomized studies have examined the relationship between surgical approach and the risk of CMS, and consequently no such data was available for inclusion. The absence of randomized trials likely reflects the ethical challenges associated with conducting such studies. Our findings are therefore based on prospective and retrospective studies, in which the observed incidence of CMS may vary. However, in the absence of randomized controlled trials, the included studies represent the currently available evidence on the association between CMS and surgical approach.

#### 4.1.2 Unadjusted odds ratio

The pooled estimates in our analysis rely on unadjusted ORs and are therefore likely confounded, warranting cautious interpretation. Possible confounders of CMS include established risk factors such as age, tumor histology, midline location, and brainstem involvement. This represents a key limitation in our analysis, as these factors may influence both CMS risk and the choice of surgical approach. The pooled estimate of all included studies indicated a non-significant increase in CMS risk with the transvermian approach, and moderate heterogeneity was observed across studies, which may have contributed to variability in effect estimates. Consequently, differences in baseline CMS risk between surgical groups may have influenced the pooled estimates, limiting causal interpretation due to potential confounding.

#### 4.1.3 Inclusion of both prospective and retrospective studies

There is a risk of bias related to the inclusion of both prospective and retrospective studies, and this heterogeneity in study design should be considered when interpreting the results, as it may affect the validity and comparability of the findings. The overall pooled effect estimate demonstrated a non-significant association between surgical approach and CMS risk. As shown in Fig. 2, all but one of the included retrospective studies reported an OR above unity, thereby affecting the overall estimate towards a positive association. In contrast, the sub-analysis of prospective studies demonstrated effect estimates distributed close to unity, with a pooled estimate suggesting no clear association between surgical approach and CMS, as shown in Fig. 3. This finding is notable, as prospective studies with pre-defined criteria are generally less prone to bias and confounding than retrospective studies. In addition, funnel plot analysis indicated a moderate degree of asymmetry, raising concerns about publication bias and overrepresentation of retrospective studies reporting positive associations and consequently, the pooled OR might be overestimated.

#### 4.1.4 CMS terminology

Different terms (CMS, CM, MSD, PFS, POSI and mutism) were treated as equivalent outcomes in this review and collectively labelled as CMS. However, these terms do not represent identical clinical entities and may not correspond precisely to CMS as defined by current consensus definitions. More inclusive definitions may capture milder cases or speech impairments unrelated to CMS, and variability in diagnostic threshold may therefore contribute to outcome heterogeneity. Some definitions place greater emphasis on speech impairment, whereas others give greater weight to psychological, cognitive, or affective impairments. For example, the ‘‘POSI’’ definition used in Grønbæk et al. (2021) is primarily focused on speech output, and the study contributed substantially to the pooled estimates in our analysis (Grønbæk et al., 2021). Similarly, the ‘‘MSD’’ definition used by Catsman-Berrevoets et al. (1999) focuses mainly on post-surgical speech behavior, including motor deficits (Catsman-Berrevoets et al., 1999). de Laurentis et al. (2022) used the ‘‘PFS’’ terminology and categorized patients as having PFS if their postoperative speech was reduced or absent, or if they showed signs of other motor – or neurobehavioral dysfunctions, thereby including cognitive and affective components (de Laurentis et al., 2022). Doxey et al. (1999), who also used the ‘‘PFS’’ definition, considered CMS as a reversible part of a broader neurological syndrome, while Onorini et al. (2022), who used the ‘‘CMS’’ definition, reported to have used the Iceland Delphi Groups diagnostic criteria (Onorini et al., 2022; Doxey et al., 1999). When different terms were collectively grouped under the CMS definition, we assumed that all definitions captured the cardinal CMS symptom (postoperative mutism or speech impairment), while differing in the extent to which associated motor-, cognitive and behavioral impairments were evaluated. This may have introduced misclassification bias as patients with similar symptom profiles may be classified differently across studies; consequently, pooling these outcomes may combine clinically and biological distinct entities and attenuate true associations. In addition, speech assessment was not performed using standardized tools which may have influenced the comparability of speech outcomes across the studies.

#### 4.1.5 Terminology of surgical approaches

Considerable variation was observed in the terminology used to describe surgical approaches across the included studies. For pragmatic reasons, these approaches were grouped as’’vermal incision’’ and’’no vermal incision’’ for calculation of ORs and were subsequently interpreted as ‘‘transvermian’’ or ‘‘telovelar’’ approaches, respectively. The purpose of the non-transvermian approach is to spare the vermis from direct injury; however, the vermis may still be subject to surgical damage other than sharp splitting. Thus, while grouping surgical terminology into’’transvermian’’ and’’telovelar’’ approaches provide a pragmatic framework for this type of review, technical details and variations in surgical technique are not captured by this categorization.

#### 4.1.6 Subanalysis of fourth ventricle tumors

Given that the fourth ventricle is a common site for pediatric brain tumors and is located in close proximity to eloquent tissues and structures frequently reported to be implicated in CMS pathophysiology, including the cerebellar vermis, deep cerebellar nuclei, and cerebellar outflow pathways (the involvement of which has been supported by recent lesion-lapping studies) a subgroup analysis of fourth ventricular tumors would have been clinically relevant but was not feasible in the present study (Toescu et al., 2021; Albazron et al., 2019). Such an analysis may have reduced patient heterogeneity and allowed for a more precise assessment of how surgical approach may influence the risk of CMS.

#### 4.1.7 Quality classification of included studies

The overall risk of bias remains substantial due to the absence of randomized controlled trials, also in studies rated as “high quality’’ based on the modified Newcastle-Ottawa Scale. This quality assessment primarily reflects reporting quality and methodological transparency, rather than true risk of bias or strength of causal inference. We therefore emphasize that these classifications should be interpreted accordingly and should not be equated with a low risk of bias.

#### 4.1.8 Strengths

Despite these limitations, this review has several strengths. It is based on a well-defined and transparent search protocol with systematic identification of relevant literature and dual-review screening. The use of a quantitative outcome measure for CMS enabled objective comparison across studies. Furthermore, the included studies represent multicenter populations, which increases generalizability. The relatively large overall sample sizes of more than 1300 patients provide a broad representation of the study population and strengthens the robustness of the pooled estimates.

### 4.2 Future perspectives

Greater standardization of diagnostic criteria and outcome definitions would likely improve the consistency and interpretability of future research regarding CMS. Efforts have been made to align terminology with the Iceland Delphi consensus paper, but no single validated diagnostic tool is currently available for the diagnosis of CMS, nor are there any standardized treatment protocols for its management (Fabozzi et al., 2022). Given the complex nature of CMS, its largely unknown etiology and thereby treatment targets, and its profound impact on affected children, there remains substantial potential to develop improved strategies for both prevention and rehabilitation.

Randomized trials comparing surgical approaches raise ethical concerns and are unlikely to be feasible in children with posterior fossa tumors. Factors such as tumor location, size, infiltration into surrounding structures, and surgeon experience may dictate the choice of surgical approach, rendering random allocation impractical and ethically questionable. Together, these factors emphasize the need for standardized definitions and grading systems, meticulous prospective study designs, and further investigation to identify modifiable risk factors for CMS more precisely.

Based on the 2024 Posterior Fossa Society meeting, Toescu et al. (2025) recommend strategies to minimize iatrogenic injury and reduce the impact of tumor resection on surrounding tissue (Toescu et al., 2025). Surgery is identified as the main modifiable risk factor for CMS, with emphasis on optimizing technique and surgical expertise. No superiority of a telovelar over a transvermian approach is demonstrated; instead, general principles are highlighted, including adequate craniotomy size for optimal exposure, avoidance of excessive suction, minimized retraction, and optimized CSF drainage. Ultrasonic aspirators may cause thermal injury and should be used cautiously, while fixed retractors are discouraged due to the risk of ischemic damage. Maintenance of hemodynamic stability and cautious hemostasis within the fourth ventricle are also emphasized, and they suggest that slightly less aggressive resection may be preferable when it preserves functionally critical structures without compromising survival. In summary, these recommendations represent general intraoperative principles aimed at minimizing tissue injury during tumor resection, rather than specific directives for preventing CMS.

Current lesion-mapping studies support minimizing vermian injury whenever possible. Albazron et al. (2019) demonstrated that CMS-related symptoms are strongly associated with lesions involving the cerebellar outflow pathways, including the deep cerebellar nuclei and superior cerebellar peduncles, with the strongest association observed in the fastigial nuclei extending into the inferior vermis (Albazron et al., 2019). Similarly, McAfee et al. (2023) identified injury to the fastigial nuclei and adjacent cerebellar cortex as strong predictors of CMS following medulloblastoma resection (McAfee et al., 2023b). Collectively, these findings highlight the relevance of the cerebellar vermis in the pathophysiology of CMS.

CMS risk may be more closely related to injury of specific cerebellar networks than to the surgical approach itself. A telovelar approach does not necessarily guarantee vermal preservation, and classifying an approach as “telovelar” or “transvermian” provides limited information regarding which structures are ultimately injured or spared during tumor resection. Together with our findings, this highlights the need for future studies on surgical risk factors for CMS to broaden their scope beyond the choice of surgical approach toward analyses incorporating lesion location, involvement of cerebellar outflow pathways, and network-based measures of injury, as current evidence suggests limited value of focusing on surgical approach alone for preventing CMS.

While no established modifiable risk factors for CMS have been identified, pharmacological interventions have been suggested as potential therapeutic options. Zolpidem has previously been reported to alleviate symptoms of CMS, and a randomized trial is currently in preparation, representing a promising avenue for the future treatment of CMS (Moshref and Mirdad, 2021; Shyu et al., 2011).

## 5 Conclusion

Our study found no statistically significant difference in CMS risk between telovelar and transvermian approaches for pediatric posterior fossa tumors; however, causal inference is limited by reliance on unadjusted ORs. Accordingly, current evidence does not support favoring one surgical approach over the other solely for the prevention of CMS. Postoperative deficits are likely driven by specific patterns of injury within cerebellar subregions and cerebellar outflow pathways that are not captured by broad surgical categorization.

## Author contributions

All authors took part in data collection, analysis and interpretation. All authors took part in preparation of the first draft of the manuscript. All authors approved the final version of the manuscript.

## Funding

This research did not receive any specific grant from funding agencies in the public, commercial, or not-for-profit sectors.

## Declaration of competing interest

The authors have no competing interests to declare.

## References

[bib1] Aarsen F.K., van Veelen-Vincent M.C., Partanen M., Catsman-Berrevoets C.E. (2022). Perioperative risk factors for long-term intelligence in children with postoperative cerebellar mutism syndrome after medulloblastoma surgery. Pediatr. Blood Cancer.

[bib2] Albazron F.M. (2019). Pediatric postoperative cerebellar cognitive affective syndrome follows outflow pathway lesions. Neurology.

[bib3] Avula S., Mallucci C., Kumar R., Pizer B. (2015). Posterior fossa syndrome following brain tumour resection: review of pathophysiology and a new hypothesis on its pathogenesis. Childs Nerv. Syst..

[bib4] Catsman-Berrevoets C.E., Van Dongen H.R., Mulder P.G., Paz y Geuze D., Paquier P.F., Lequin M.H. (1999). Tumour type and size are high risk factors for the syndrome of "cerebellar" mutism and subsequent dysarthria. J. Neurol. Neurosurg. Psychiatry.

[bib5] Cobourn K. (2020). Cerebellar mutism syndrome: current approaches to minimize risk for CMS. Childs Nerv. Syst..

[bib7] de Laurentis C. (2022). Posterior fossa syndrome in a population of children and young adults with medulloblastoma: a retrospective, multicenter Italian study on incidence and pathophysiology in a histologically homogeneous and consecutive series of 136 patients. J. Neuro Oncol..

[bib6] De Smet H.J., Catsman-Berrevoets C., Aarsen F., Verhoeven J., Mariën P., Paquier P.F. (2012). Auditory-perceptual speech analysis in children with cerebellar tumours: a long-term follow-up study. Eur. J. Paediatr. Neurol..

[bib8] Di Rocco C., Chieffo D., Frassanito P., Caldarelli M., Massimi L., Tamburrini G. (2011). Heralding cerebellar mutism: evidence for pre-surgical language impairment as primary risk factor in posterior fossa surgery. Cerebellum.

[bib9] Doxey D., Bruce D., Sklar F., Swift D., Shapiro K. (1999). Posterior fossa syndrome: identifiable risk factors and irreversible complications. Pediatr. Neurosurg..

[bib10] Erşahin Y., Yararbas U., Duman Y., Mutluer S. (2002). Single photon emission tomography following posterior fossa surgery in patients with and without mutism. Childs Nerv. Syst..

[bib11] Fabozzi F. (2022). Cerebellar mutism syndrome: from pathophysiology to rehabilitation. Front. Cell Dev. Biol..

[bib12] Gora N.K., Gupta A., Sinha V.D. (2017). Cerebellar mutism syndrome following midline posterior Fossa Tumor resection in children: an institutional experience. J. Pediatr. Neurosci..

[bib13] Grill J. (2004). Critical risk factors for intellectual impairment in children with posterior fossa tumors: the role of cerebellar damage. J. Neurosurg..

[bib14] Grønbæk J.K. (2021). Postoperative speech impairment and surgical approach to posterior fossa tumours in children: a prospective European multicentre cohort study. Lancet Child Adolesc. Health.

[bib15] Gudrunardottir T. (2016). Consensus paper on post-operative pediatric cerebellar mutism syndrome: the Iceland Delphi results. Childs Nerv. Syst..

[bib16] Hanaei S. (2024). Telovelar vs. Transvermian approach for the fourth ventricle tumors: a systematic review and meta-analysis. Clin. Neurol. Neurosurg..

[bib17] Haratek K. (2023). Predictors of postoperative complications and functional outcomes in pediatric patients with surgically treated fourth ventricle tumors. Acta Neurochir..

[bib18] Korah M.P. (2010). Incidence, risks, and sequelae of posterior fossa syndrome in pediatric medulloblastoma. Int. J. Radiat. Oncol. Biol. Phys..

[bib19] McAfee S.S. (2023). Cerebellar mutism is linked to midbrain volatility and desynchronization from speech cortices. Brain.

[bib20] McAfee S.S. (2023). Fastigial nuclei surgical damage and focal midbrain disruption implicate PAG survival circuits in cerebellar mutism syndrome. Neuro Oncol..

[bib21] McAfee S.S. (2024). Secondary cerebro-cerebellar and intra-cerebellar dysfunction in cerebellar mutism syndrome. Neuro Oncol..

[bib22] McMillan H.J., Keene D.L., Matzinger M.A., Vassilyadi M., Nzau M., Ventureyra E.C. (2009). Brainstem compression: a predictor of postoperative cerebellar mutism. Childs Nerv. Syst..

[bib23] Moshref R., Mirdad A. (2021). Cerebellar mutism treated successfully with zolpidem in a patient with learning difficulties. Cureus.

[bib24] Onorini N. (2022). The clinical and prognostic impact of the choice of surgical approach to fourth ventricular tumors in a single-center, single-surgeon cohort of 92 consecutive pediatric patients. Front. Oncol..

[bib25] Pettersson S.D., Kitlinski M., Miękisiak G., Ali S., Krakowiak M., Szmuda T. (2022). Risk factors for postoperative cerebellar mutism syndrome in pediatric patients: a systematic review and meta-analysis. J. Neurosurg. Pediatr..

[bib26] Renne B. (2020). Cerebellar mutism after posterior fossa tumor resection in children: a multicenter international retrospective study to determine possible modifiable factors. Childs Nerv. Syst..

[bib27] Rudolph S. (2023). Cognitive-affective functions of the cerebellum. J. Neurosci..

[bib28] Schmidt S. (2023). Cerebellar mutism syndrome after posterior Fossa tumor surgery in Children-A retrospective single-center study. World Neurosurg..

[bib29] Severo Bem L., Gemir J., Cysneiros R.R.M., Azevedo H.C. (2021). The understanding of pediatric akinetic mutism. Cureus.

[bib30] Shyu C. (2011). Novel use of zolpidem in cerebellar mutism syndrome. J. Pediatr. Hematol. Oncol..

[bib32] Toescu S.M. (2021). Fourth ventricle tumors in children: complications and influence of surgical approach. J. Neurosurg. Pediatr..

[bib33] Toescu S.M. (2025). Toward reducing the risk of cerebellar mutism syndrome: consensus statement from the posterior fossa society. J. Neurosurg. Pediatr..

[bib31] Toescu S.M., Hales P.W., Aquilina K., Clark C.A. (2018). Quantitative MRI in post-operative paediatric cerebellar mutism syndrome. Eur. J. Radiol..

[bib36] Wells E.M. (2010). Postoperative cerebellar mutism syndrome following treatment of medulloblastoma: neuroradiographic features and origin. J. Neurosurg. Pediatr..

[bib35] Wells G. (2000). The Newcastle–Ottawa Scale (NOS) for Assessing the Quality of Non-randomized Studies in Meta-Analysis.

[bib34] Wells E.M., Packer R.J. (2015). Pediatric brain tumors. Continuum (Minneap Minn).

[bib37] Wibroe M., Ingersgaard M.V., Larsen H.B., Juhler M., Piil K. (2021). Living with the cerebellar mutism syndrome: long-term challenges of the diagnosis. Acta Neurochir..

[bib38] Wisoff J.H., Epstein F.J. (1984). Pseudobulbar palsy after posterior fossa operation in children. Neurosurgery.

[bib39] Yang W. (2023). Male predisposition in cerebellar mutism syndrome: a cohort study. Cerebellum.

