# Supplementary material for: Does the surgical route matter in cerebellar mutism syndrome? Comparing transvermian and telovelar approaches for pediatric posterior fossa tumors
Source: Brain Spine. 2026 Jul 27;6:106168. doi: 10.1016/j.bas.2026.106168 (PMC13450502; doi:10.1016/j.bas.2026.106168)
Supplement: Supplementary file 1 [file mmc1.docx]

**Supplementary material**

**Title**

Does the surgical route matter in cerebellar mutism syndrome? Comparing transvermian and telovelar approaches for pediatric posterior fossa tumors.

**Journal**

Brain and Spine

**Author list**

Merle Egeborg^1^, BM

Rebekka Sarup^2^, BM

Marianne Juhler^1,3*^, Professor, Dr.Med

Aske Foldbjerg Laustsen^1,2*^, MD

*Shared senior authorship

**Affiliation**

^1^Department of Neurosurgery, Rigshospitalet, Copenhagen, Denmark

^2^Department of Pediatrics and Adolescent Medicine, Rigshospitalet, Copenhagen, Denmark

^3^Department of Neurosurgery, Aarhus University Hospital, Aarhus, Denmark

**Corresponding authors**

Merle Egeborg (e-mail: [jhb871@alumni.ku.dk](mailto:jhb871@alumni.ku.dk))

Aske Foldbjerg Laustsen (e-mail: [aske.foldbjerg.laustsen@regionh.dk](mailto:aske.foldbjerg.laustsen@regionh.dk))

Postal address

Department of Neurosurgery, Rigshospitalet

Blegdamsvej 9, 2100 Copenhagen, Denmark

**Figure S1** PubMed search strategy

The search strategy was developed to cover the three main aspects of the PICO framework.

The 3 aspects were combined with ”AND’’

| **Aspect 1** | **Aspect 2** | **Aspect 3** |
| --- | --- | --- |
| Posterior fossa syndrome | Neurosurgical procedures | Child |
| OR | OR | OR |
| Mutism | Transvermian  Telovelar  Craniectomy  Posterior fossa surgery | Young  Youth  Infant  Toddler  Adolescent  Pediatric  Paediatric |

Search number,Query,Sort By,Filters,Search Details,Results,Time

5,((((posterior fossa syndrome) OR (mutism)) AND ((neurosurgical procedures) OR (transvermian) OR (telovelar) OR (craniectomy) OR (fossa posterior surgery))) AND (Child OR Young OR infant OR toddler OR adolescent OR pediatric OR paediatric)) AND (1900/01/01:2024/03/30[dp]),Most Recent,,"(((""posterior""[All Fields] OR ""posteriors""[All Fields]) AND (""fossa""[All Fields] OR ""fossae""[All Fields] OR ""fossas""[All Fields]) AND (""syndrom""[All Fields] OR ""syndromal""[All Fields] OR ""syndromally""[All Fields] OR ""syndrome""[MeSH Terms] OR ""syndrome""[All Fields] OR ""syndromes""[All Fields] OR ""syndrome s""[All Fields] OR ""syndromic""[All Fields] OR ""syndroms""[All Fields])) OR (""mutism""[MeSH Terms] OR ""mutism""[All Fields] OR ""mutisms""[All Fields])) AND (""neurosurgical procedures""[MeSH Terms] OR (""neurosurgical""[All Fields] AND ""procedures""[All Fields]) OR ""neurosurgical procedures""[All Fields] OR ""transvermian""[All Fields] OR ""telovelar""[All Fields] OR (""craniotomy""[MeSH Terms] OR ""craniotomy""[All Fields] OR ""craniectomies""[All Fields] OR ""craniectomy""[All Fields]) OR ((""fossa""[All Fields] OR ""fossae""[All Fields] OR ""fossas""[All Fields]) AND (""posterior""[All Fields] OR ""posteriors""[All Fields]) AND (""surgery""[MeSH Subheading] OR ""surgery""[All Fields] OR ""surgical procedures, operative""[MeSH Terms] OR (""surgical""[All Fields] AND ""procedures""[All Fields] AND ""operative""[All Fields]) OR ""operative surgical procedures""[All Fields] OR ""general surgery""[MeSH Terms] OR (""general""[All Fields] AND ""surgery""[All Fields]) OR ""general surgery""[All Fields] OR ""surgery s""[All Fields] OR ""surgerys""[All Fields] OR ""surgeries""[All Fields]))) AND (""child""[MeSH Terms] OR ""child""[All Fields] OR ""children""[All Fields] OR ""child s""[All Fields] OR ""children s""[All Fields] OR ""childrens""[All Fields] OR ""childs""[All Fields] OR (""young""[All Fields] OR ""youngs""[All Fields]) OR (""infant""[MeSH Terms] OR ""infant""[All Fields] OR ""infants""[All Fields] OR ""infant s""[All Fields]) OR (""toddler""[All Fields] OR ""toddler s""[All Fields] OR ""toddlers""[All Fields]) OR (""adolescences""[All Fields] OR ""adolescency""[All Fields] OR ""adolescent""[MeSH Terms] OR ""adolescent""[All Fields] OR ""adolescence""[All Fields] OR ""adolescents""[All Fields] OR ""adolescent s""[All Fields]) OR (""paediatrics""[All Fields] OR ""pediatrics""[MeSH Terms] OR ""pediatrics""[All Fields] OR ""paediatric""[All Fields] OR ""pediatric""[All Fields]) OR (""paediatrics""[All Fields] OR ""pediatrics""[MeSH Terms] OR ""pediatrics""[All Fields] OR ""paediatric""[All Fields] OR ""pediatric""[All Fields])) AND 1900/01/01:2024/09/10[Date - Publication]",767,01:23:53

4,1900/01/01:2024/09/10[dp],Most Recent,,1900/01/01:2024/09/10[Date - Publication],"36,982,510",01:23:27

3,Child OR Young OR infant OR toddler OR adolescent OR pediatric OR paediatric,Most Recent,,"""child""[MeSH Terms] OR ""child""[All Fields] OR ""children""[All Fields] OR ""child s""[All Fields] OR ""children s""[All Fields] OR ""childrens""[All Fields] OR ""childs""[All Fields] OR ""young""[All Fields] OR ""youngs""[All Fields] OR ""infant""[MeSH Terms] OR ""infant""[All Fields] OR ""infants""[All Fields] OR ""infant s""[All Fields] OR ""toddler""[All Fields] OR ""toddler s""[All Fields] OR ""toddlers""[All Fields] OR ""adolescences""[All Fields] OR ""adolescency""[All Fields] OR ""adolescent""[MeSH Terms] OR ""adolescent""[All Fields] OR ""adolescence""[All Fields] OR ""adolescents""[All Fields] OR ""adolescent s""[All Fields] OR ""paediatrics""[All Fields] OR ""pediatrics""[MeSH Terms] OR ""pediatrics""[All Fields] OR ""paediatric""[All Fields] OR ""pediatric""[All Fields] OR ""paediatrics""[All Fields] OR ""pediatrics""[MeSH Terms] OR ""pediatrics""[All Fields] OR ""paediatric""[All Fields] OR ""pediatric""[All Fields]","6,148,297",01:21:25

2,(neurosurgical procedures) OR (transvermian) OR (telovelar) OR (craniectomy) OR (fossa posterior surgery),Most Recent,,"""neurosurgical procedures""[MeSH Terms] OR (""neurosurgical""[All Fields] AND ""procedures""[All Fields]) OR ""neurosurgical procedures""[All Fields] OR ""transvermian""[All Fields] OR ""telovelar""[All Fields] OR (""craniotomy""[MeSH Terms] OR ""craniotomy""[All Fields] OR ""craniectomies""[All Fields] OR ""craniectomy""[All Fields]) OR ((""fossa""[All Fields] OR ""fossae""[All Fields] OR ""fossas""[All Fields]) AND (""posterior""[All Fields] OR ""posteriors""[All Fields]) AND (""surgery""[MeSH Subheading] OR ""surgery""[All Fields] OR ""surgical procedures, operative""[MeSH Terms] OR (""surgical""[All Fields] AND ""procedures""[All Fields] AND ""operative""[All Fields]) OR ""operative surgical procedures""[All Fields] OR ""general surgery""[MeSH Terms] OR (""general""[All Fields] AND ""surgery""[All Fields]) OR ""general surgery""[All Fields] OR ""surgery s""[All Fields] OR ""surgerys""[All Fields] OR ""surgeries""[All Fields]))","244,101",01:20:04

1,(posterior fossa syndrome) OR (mutism),Most Recent,,"((""posterior""[All Fields] OR ""posteriors""[All Fields]) AND (""fossa""[All Fields] OR ""fossae""[All Fields] OR ""fossas""[All Fields]) AND (""syndrom""[All Fields] OR ""syndromal""[All Fields] OR ""syndromally""[All Fields] OR ""syndrome""[MeSH Terms] OR ""syndrome""[All Fields] OR ""syndromes""[All Fields] OR ""syndrome s""[All Fields] OR ""syndromic""[All Fields] OR ""syndroms""[All Fields])) OR (""mutism""[MeSH Terms] OR ""mutism""[All Fields] OR ""mutisms""[All Fields])","5,379",01:19:12

**Figure S2** EMBASE search strategy

Embase <1974 to 2024 September 10>

1	mutism/	3515

2	posterior fossa syndrome.ab,kf,ti.	242

3	1 or 2	3655

4	exp neurosurgery/	322539

5	transvermian.ab,kf,ti.	56

6	telovelar.ab,kf,ti.	145

7	craniotomy.ab,kf,ti.	24731

8	craniectomy.ab,kf,ti.	8164

9	posterior fossa surgery.ab,kf,ti.	677

10	4 or 5 or 6 or 7 or 8 or 9	326849

11	3 and 10	423

12	(mutism or mute*).ab,kf,ti.	8737

13	1 or 2 or 12	10138

14	10 and 13	562

15	juvenile/ or exp adolescent/ or exp child/	4172492

16	exp pediatrics/	132007

17	(child* or young or youth or infant or infants or toddler* or p?ediatric*).ab,kf,ti.	3494067

18	15 or 16 or 17	5404675

19	10 and 13 and 18	358

20	limit 19 to conference abstract	76

21	19 not 20	282

| **Table S1 Newcastle-Ottawa quality scoring of included studies** | | | | | | | | | | |
| --- | --- | --- | --- | --- | --- | --- | --- | --- | --- | --- |
| Included studies | Selection | | | | Comparability* | Exposure | | | Overall quality | |
| Cohort | Representativeness of exposed cohort | Selection of non-exposed cohort | Ascertainment of exposure | Outcome not present at start of study |  | Assessment of outcome | Appropriate follow-up | Adequacy of follow-up | Score | Rating |
| Catsman-Berrevoets et al. | 1 | 1 | 1 | 1 | 0 | 1 | 1 | 1 | 7/8 | High |
| Cobourn et al. | 1 | 1 | 1 | 0 | 0 | 1 | 1 | 1 | 6/8 | Moderate |
| De Laurentis et al. | 1 | 1 | 1 | 0 | 1 | 1 | 1 | 1 | 7/8 | High |
| De Smet et al. | 1 | 1 | 1 | 0 | 1 | 1 | 1 | 1 | 7/8 | High |
| Di Rocco et al. | 1 | 1 | 1 | 1 | 0 | 1 | 1 | 1 | 7/8 | High |
| Doxey et al. | 1 | 1 | 1 | 0 | 0 | 1 | 1 | 1 | 6/8 | Moderate |
| Erşahin et al | 1 | 1 | 1 | 0 | 0 | 0 | 1 | 1 | 5/8 | Moderate |
| Gora et al. | 1 | 1 | 1 | 1 | 0 | 1 | 1 | 1 | 7/8 | High |
| Grill et al | 1 | 1 | 1 | 1 | 0 | 0 | 1 | 1 | 6/8 | Moderate |
| Grønbæk et al. | 1 | 1 | 1 | 1 | 1 | 1 | 1 | 1 | 8/8 | High |
| Haratek et al | 1 | 1 | 1 | 0 | 0 | 1 | 1 | 1 | 6/8 | Moderate |
| McMillan et al. | 1 | 1 | 1 | 0 | 0 | 0 | 1 | 1 | 5/8 | Moderate |
| Onorini et al. | 1 | 1 | 1 | 0 | 0 | 1 | 1 | 1 | 6/8 | Moderate |
| Schmidt et al | 1 | 1 | 1 | 1 | 1 | 1 | 1 | 1 | 8/8 | High |
| Toescu et al. | 1 | 1 | 1 | 0 | 1 | 1 | 1 | 1 | 7/8 | High |
| Yang et al. | 1 | 1 | 1 | 1 | 0 | 1 | 1 | 1 | 7/8 | High |
| *Modified to categorize whether surgical approach on the risk of CMS was a primary outcome of the included study | | | | | | | | | | |

**Figure S3** Funnel plot of publication bias

Colors in the plot indicate study design: red = retrospective study, blue = prospective study and purple = mixed cohort of prospective and retrospective.

**Figure S4** Forest plot without studies of zero observations columns

Colors in the plot indicate study design: red = retrospective study, blue = prospective study and purple = mixed cohort of prospective and retrospective.
